# Influenza vaccine effectiveness against hospitalisation due to laboratory-confirmed influenza in children in England in the 2015–2016 influenza season – a test-negative case–control study

**DOI:** 10.1017/S0950268819000876

**Published:** 2019-05-30

**Authors:** N. L. Boddington, F. Warburton, H. Zhao, N. Andrews, J. Ellis, M. Donati, R. G. Pebody

**Affiliations:** 1 Public Health England, London, UK; 2 Public Health England National Infection Service South West and Severn Infection Sciences, University Hospital Bristol, Bristol, UK

**Keywords:** Children's vaccines, influenza (seasonal), influenza, vaccination (immunisation), vaccine effectiveness

## Abstract

England has recently started a new paediatric influenza vaccine programme using a live-attenuated influenza vaccine (LAIV). There is uncertainty over how well the vaccine protects against more severe end-points. A test-negative case–control study was used to estimate vaccine effectiveness (VE) in vaccine-eligible children aged 2–16 years of age in preventing laboratory-confirmed influenza hospitalisation in England in the 2015–2016 season using a national sentinel laboratory surveillance system. Logistic regression was used to estimate the VE with adjustment for sex, risk-group, age group, region, ethnicity, deprivation and month of sample collection. A total of 977 individuals were included in the study (348 cases and 629 controls). The overall adjusted VE for all study ages and vaccine types was 33.4% (95% confidence interval (CI) 2.3–54.6) after adjusting for age group, sex, index of multiple deprivation, ethnicity, region, sample month and risk group. Risk group was shown to be an important confounder. The adjusted VE for all influenza types for the live-attenuated vaccine was 41.9% (95% CI 7.3–63.6) and 28.8% (95% CI −31.1 to 61.3) for the inactivated vaccine. The study provides evidence of the effectiveness of influenza vaccination in preventing hospitalisation due to laboratory-confirmed influenza in children in 2015–2016 and continues to support the rollout of the LAIV childhood programme.

## Introduction

In 2013, the United Kingdom (UK) started the introduction of a paediatric influenza vaccination programme following recommendations of the Joint Committee on Vaccination and Immunisation (JCVI) in 2012 [1]. The aim of this programme is to ultimately offer annual influenza vaccination to all children 2–11 years of age to both directly protect them, and by reducing their rate of infection, indirectly protect others in the community who may be at higher risk of severe disease following infection [1]. The programme initially targeted all 2 and 3 year olds in 2013/14 and has been incrementally extended in subsequent seasons to further age groups. Once it has been extended to include all 2–11 years olds the programme will be paused and evaluated.

Through this programme, healthy children are offered a single dose live-attenuated influenza vaccine (LAIV) which is administered intranasally. The live-attenuated vaccine was recommended compared with the injectable, inactivated vaccine due to apparent higher effectiveness in children, potential to provide cross-protection against poorly vaccine-virus matched strains, higher acceptability amongst children, their parents and carers and possible longer-term immunological advantages [2].

The 2015–2016 season was the third season of the introduction of this paediatric influenza vaccination programme. All healthy children aged 2–4 years of age, together with children of school years 1 and 2 (ages 5 and 6 years) across England were offered quadrivalent LAIV [3]. In addition, children aged 2–16 years in a clinical risk group were also offered LAIV where not contraindicated, with the remainder offered quadrivalent inactivated vaccine.

The 2015–2016 influenza season started late in England and peaked in week 11 [4]. Comparatively large numbers of hospitalisations and admissions to intensive care units, particularly in younger adults were seen [4]. The season was dominated by circulation of influenza A(H1N1)pdm09, which was well matched to the A(H1N1)pdm09 2015–16 vaccine strain, and later by influenza B, predominantly of the B/Victoria lineage, which was not included in the 2015–2016 trivalent inactivated influenza vaccine [4]. The end-of-season vaccine effectiveness (VE) against laboratory-confirmed influenza infection in primary care in children for LAIV was moderately good against all influenza types (57.6%, 95% confidence interval (CI) 25.1–76.0) with moderate, but non-significant VE for influenza A(H1N1)pdm09 (41.5%, 95% CI −8.5 to 68.5) and high VE for influenza B (81.4%, 95% CI 39.6–94.3) [5]. Similar LAIV effectiveness results in children were also seen in the first two seasons of the programme [6]. However these findings for LAIV in children contrast those reported by the US Center for Disease Control and Prevention (CDC) in 2015–2016 who found an overall VE of only 5% (95% CI −47 to 39) in 2–17 years old children with a VE against influenza A(H1N1)pdm09 of −19% (95% CI −113 to 33) [7]. These findings led to the recommendation that LAIV should not be used in the USA by the Advisory Committee on Immunisation Practice (ACIP) [8].

For the first time in 2015–2016, the UK also published data on the effectiveness of LAIV against more severe disease in a study using the screening method [9]. This study found evidence that the LAIV was effective in preventing laboratory-confirmed influenza hospitalisation in children 2–6 years of age in England in 2015–2016 [9]. The screening method can be a useful study design for estimating VE rapidly and inexpensively as it uses routinely available population data when there is a lack of suitable controls. Despite this, the screening method has a number of potential limitations; most notably the cases may arise from a population that differs from that used to determine vaccine uptake rates and the inability to adjust for important but unmeasured confounders.

The aim of this enhanced surveillance project is to evaluate influenza VE in children of 2–16 years in England in 2015–2016 in protecting against laboratory-confirmed infection resulting in hospitalisation using the alternative test-negative case–control (TNCC) design.

## Methods

### Study design

The test-negative design is a particular type of case–control study. Using this study design participants are recruited if they meet a certain clinical case definition and are tested for the infection in question. The odds of vaccination are then compared between those testing positive *vs.* those testing negative to estimate VE. A TNCC study was used to estimate the VE in vaccine-eligible children aged 2–16 years in preventing laboratory-confirmed influenza hospitalisation in England in the 2015–2016 season.

### Setting and participants

Cases and controls were both identified from the Respiratory DataMart System. This is a national sentinel laboratory surveillance system which records details of individuals tested for suspect influenza infection. Suspect cases are tested for influenza, respiratory syncytial virus, rhinovirus, parainfluenza 1–4 and human metapneumovirus using reverse transcription real-time polymerase chain reaction (rRT-PCR), and adenovirus using real-time PCR on respiratory samples by 14 laboratories located across England [10]. The most common sample types are nasopharyngeal aspirate, tracheal secretion and nasal and throat swabs. On average the total number of samples tested each year from these participating laboratories is 70 000 per year. Those testing positive for other respiratory viruses were not excluded from the analysis. Cases and controls were recruited during the 2015–2016 influenza season between week 40 of 2015 and week 20 of 2016.

### Participants

#### Cases

A case was defined as an individual with laboratory-confirmed influenza A or/and B infection (confirmed by RT-PCR) with a specimen date from week 40 of 2015 to week 20 of 2016 aged between 2 and 16 years old (on 31 August 2015) and resident in England.

#### Controls

A control was defined as an individual who was tested for influenza infection, with a specimen date from week 40 of 2015 to week 20 of 2016 and tested negative for influenza infection (by RT-PCR) aged 2–16 years and resident in England.

Controls were group-matched to cases by age group (2–4, 5–8, 9–11, 12–17) and week of sample with up to three controls randomly selected per case within these groups. If fewer than three controls were available then all available controls were selected in that strata. Estimated population figures by age group and region are provided in Table 1.

**Table 1.** 2015 mid-year population estimates by age group and region in England [11]

	2–4	5–6	7–8	9–11	12–16
North	289,409	190,150	187,947	264,656	425,787
South	271,116	178,936	177,776	248,677	408,070
Midlands and East	321,320	210,092	207,860	292,570	474,138
London	194,446	119,287	115,522	154,752	236,308

### Variables

Demographic details of cases and controls from the DataMart system were used to identify the primary care (general) practitioners (GPs) of these children, using the Patient Demographic Service (PDS) system. Any individuals not identifiable by the PDS system as being registered with a GP or as not resident in England were excluded from the study. Postal questionnaires were then sent to the identified GPs to ascertain whether the child had received influenza vaccination during the 2015–2016 season and if so, the vaccination date and whether the vaccine was administered by injection or intranasally and whether they had been vaccinated in the previous season. Information on whether the child was in a clinical risk group for vaccination was also obtained from the GPs.

The outcome of interest was laboratory-confirmed influenza infection (confirmed with RT-PCR through the Respiratory DataMart system) and the exposure was vaccination against influenza during the 2015–2016 influenza season.

Data on a number of potential *a priori* confounders were collected including age group, sex, ethnicity, region, index of multiple deprivation (IMD) and month of sample collection. These have been shown to confound the vaccination-influenza effect [12]. Risk group was also explored as a possible confounder since the presence of certain medical condition may increase a person's risk of severe influenza as well as being an eligibility criterion for free vaccination [12]. Risk groups included were those as defined in the UK Immunisation against Infectious Disease Book (‘Green Book’) [13] and individuals belonging to one or more of these risk groups were categorised as being in a risk group.

The 2015 IMD decile for the child was based on the place of residence (1–10, where 1 is the most deprived and 10 the least deprived) [14]. Ethnic group was assigned using Onomap software [15]. The Onomap software assigned each study subject into one of the UK 2001 census ethnic groups which were then grouped into the following categories: White, Asian, Black and Other ethnicity.

### Statistical methods

A child was considered vaccinated if they received at least one dose of influenza vaccine at least 14 days before the child's date of reported symptom onset, the assumed minimum time period for the child to achieve maximum protection. Due to a large proportion of individuals missing the dates of onset, the sample date minus 4 days was taken as a proxy onset date, which was the median time amongst those in whom the information was available.

If the child was vaccinated less than 14 days before onset, had an unknown vaccination record, or the vaccine was given less than 14 days before the onset of symptoms then the child was excluded from the analysis. A child was considered unvaccinated if they were reported to have received no vaccine. Where the date of vaccination was missing, the median date of vaccination amongst the vaccinated cases and controls where known was taken (31 October 2015).

#### Descriptive analysis

The characteristics of cases and controls are described and compared by baseline characteristics including sex, age, IMD quintile, ethnicity and region of residence, using the χ^2^ test or Fisher's exact test as appropriate.

#### Crude and adjusted vaccine effectiveness

Logistic regression was used to calculate the unadjusted odds ratios (OR) for influenza vaccination in cases compared with controls, with a 95% CI, with influenza test result as the outcome and influenza vaccination status as the predictor. VE is defined as (1 − OR) × 100.

Adjusted estimates were estimated using sex, age group, region, ethnicity, deprivation and month of sample collection. Risk group was also investigated as a potential confounding variable.

Adjusted VE estimates were calculated overall and also examined by type of influenza (influenza A, influenza A(H1N1)pdm09 and influenza B), type of vaccination (intranasal, intramuscular), age group (2–7 and 8–16) and prior vaccination.

All statistical analyses were carried out in Stata version 13.1 (StataCorp., USA).

### Governance

This work was undertaken as a routine public health function to monitor vaccination programmes; Public Health England (PHE) holds permissions under Section 251 (Regulation 3) of the 2002 Health Service (Control of Patient Information) Regulations to process patient identifiable information without patient consent as part of monitoring and evaluation of national vaccination programmes.

## Results

### Descriptive analysis

**Fig. 1.**
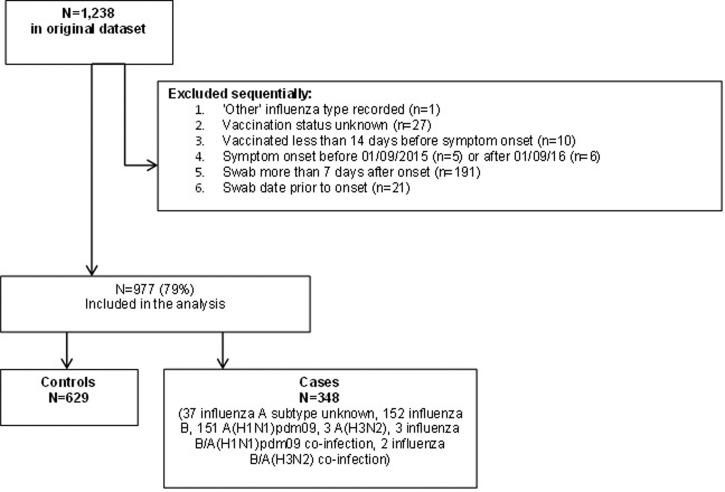
Patient inclusion and exclusion criteria.

There were a total of 1238 children aged between 2 and 16 years (on 31 August 2015) reported to DataMart, who were hospitalised between week 40 of 2015 and week 20 of 2016 and tested for influenza infection. Two-hundred and fifty-six individuals were excluded (20.7%). These individuals were excluded due to having ‘other’ recorded as the influenza type (*n* = 1), unknown vaccination status (*n* = 27), due to being vaccinated less than 14 days before symptom onset (*n* = 10), symptom onset either before or after the study period (*n* = 11) and having a swab taken either prior to onset or more than 7 days after onset (*n* = 201). The remaining 977 individuals were selected for analysis (Fig. 1). There were 34 individuals with unknown vaccination dates however the median date of vaccination from those where the information was known was used instead. The median date was 31 October 2015 which was assumed to be valid since influenza activity occurred late during the season, peaking around week 11, as well as the vaccination programme being completed by end of January. It was thus likely cases would have been fully immunised prior to the onset of influenza activity.

Of the 977 included individuals, there were 348 cases and 629 controls. Of the cases, 151 (43.4%) tested positive for influenza A(H1N1)pdm09, 152 for influenza B (43.7%), 37 for influenza A (subtype unspecified) (10.5%), three for influenza A(H3N2) (0.9%) and five were co-infections (1.4%) (Table 2).

**Table 2.** Characteristics of influenza cases (*n* = 348) and controls (*n* = 629)

	Cases (%) (*n* = 348)	Controls (%) (*n* = 629)	Total (*n* = 977)	*P*-value
Age group (years)				
2–4	156 (44.8)	310 (49.3)	466	0.556
5–6	50 (14.4)	81 (12.9)	131	
7–8	46 (13.2)	68 (10.8)	114	
9–11	36 (10.3)	56 (8.9)	92	
12–16	60 (17.2)	114 (18.1)	174	
Sex				
Female	158 (45.4)	308 (49.0)	466	0.285
Male	190 (54.6)	321 (51.0)	511	
Ethnic group				
White	250 (71.8)	495 (78.7)	745	0.028
Asian	57 (16.4)	72 (11.4)	129	
Black	5 (1.4)	15 (2.4)	20	
Other	31 (8.9)	38 (6.0)	69	
Missing	5 (1.4)	9 (1.4)	14	
IMD				
1	64 (18.4)	124 (19.7)	188	0.408
2	57 (16.4)	67 (10.7)	124	
3	41 (11.8)	76 (12.1)	117	
4	38 (10.9)	63 (10.0)	101	
5	23 (6.6)	53 (8.4)	76	
6	17 (4.9)	46 (7.3)	63	
7	29 (8.3)	55 (8.7)	84	
8	28 (8.0)	52 (8.3)	80	
9	24 (6.9)	46(7.3)	70	
10	27 (7.8)	47 (7.5)	74	
Month of sample collection				
October	2 (0.6)	9 (1.4)	11	<0.0001
November	1 (0.3)	18 (2.9)	19	
December	13 (3.7)	77 (12.2)	90	
January	49 (14.1)	96 (15.3)	145	
February	75 (21.6)	152 (24.2)	227	
March	150 (43.1)	170 (27.0)	320	
April	47 (13.5)	72 (11.4)	119	
May	11 (3.2)	35 (5.6)	46	
Risk group				
Yes	101 (29.0)	333 (52.9)	434	<0.0001
No	132 (37.9)	251 (39.9)	383	
Missing	115 (33.0)	45 (7.2)	160	
Regions				
North	143 (41.1)	168 (26.7)	311	<0.0001
South	88 (25.3)	158 (25.1)	246	
Midlands + East	91 (26.1)	211 (33.5)	302	
London	26 (7.5)	92 (14.6)	118	
Vaccination status				
Vaccinated	78 (26.3)	219 (73.7)	297	<0.0001
Not vaccinated	270 (39.7)	410 (60.3)	680	
Vaccination status (by route)				
Intramuscular	27 (7.8)	78 (12.4)	105	0.869
Intranasal	46 (13.2)	127 (20.2)	173	
Missing	5 (1.4)	15 (2.4)	20	
Influenza type/subtype				
Influenza A/H1N1pdm09	151 (43.4)			
Influenza A/H3N2	3 (0.9)			
Influenza A unknown subtype	37 (10.5)			
Influenza B	152 (43.7)			
Co-infection (3 influenza A(H1N1)pdm09–B, 2 influenza A(H3N2)–B)	5 (1.4)			

The demographic characteristics of the cases and controls are summarised in Table 2. The majority of recruited individuals were between 2 and 4 years of age (47.7%) and there was a roughly equal ratio of males and females included in the study (52.3% and 47.7% respectively). Where known, the majority of the participants were of White ethnicity (77.4%) followed by Asian ethnicity (13.4%). Ethnicity was missing for 14 individuals. Data on the risk group status was unknown or missing for 16.4% of individuals. Where known, a large proportion of all study individuals had a risk factor (53.1%). A greater proportion of controls had a risk factor (52.9%) compared with cases with a risk factor (29.0%).

Almost one-third of the individuals included in the study were vaccinated against influenza in 2015–2016 (30.4%) and the majority of vaccinated individuals (62.2%), where information was known, received the vaccine intranasally (LAIV). Information on the route of vaccination was missing for 19 individuals who were excluded from VE estimates stratified by route.

Positivity rates between cases and controls differed significantly by the ethnic group, month of sample collection, risk group status, region and vaccination status, but not by age group, sex, IMD and route of vaccination (Table 2). Whilst there was no significant difference in positivity rates by IMD deciles (*P* = 0.408), there was an increasing number of individuals included in the study with increasing deprivation.

### Vaccine effectiveness estimates

Explanatory variables were added to the model in a step-wise manner (Table 3). Risk factor was the only confounder for the vaccine effects which changed the point estimates by more than 5%, however all *a priori* confounders were incorporated into the final multivariable model (Table 4).

**Table 3.** Stepwise addition of explanatory variables and respective adjusted VE estimates

Possible confounding variable	VE estimate (95% CI)
Unadjusted	45.9 (26.9–60.0)
+ Age group	45.5 (26.3–59.7)
+ Age group and sex	46.1 (27.0–60.1)
+ Age group, sex and IMD	45.5 (26.1–59.8)
+ Age group, sex, IMD and ethnicity	45.8 (26.4–60.1)
+ Age group, sex, IMD, ethnicity and region	47.3 (28.0–61.4)
+ Age group, sex, IMD, ethnicity, region and month	46.6 (26.4–61.3)
+ Age group, sex, IMD, ethnicity, region, month and risk group (final model)	33.4 (2.3–54.6)

**Table 4.** Vaccine uptake in cases and controls by explanatory variables

	Cases (%) (*n* = 348)		Controls (%) (*n* = 629)		Total (*n* = 977)
	Vacc (%)	Unvacc (%)	Vacc (%)	Unvacc (%)	
Age Group					
2–4	43 (27.6)	113 (72.4)	111 (35.8)	199 (64.2)	466
5–6	13 (26.0)	37 (74.0)	35 (43.2)	46 (56.8)	131
7–8	4 (8.7)	42 (91.3)	19 (27.9)	49 (72.1)	114
9–11	7 (19.4)	29 (80.6)	15 (26.8)	41 (73.2)	92
12–16	11 (18.3)	49 (81.7)	39 (34.2)	75 (65.8)	174
Sex					
Female	34 (21.5)	124 (78.5)	96 (31.2)	212 (68.8)	466
Male	44 (23.2)	146 (76.8)	123 (38.3)	198 (61.7)	511
Ethnic Group					
White	14 (24.6)	43 (75.4)	25 (34.7)	47 (65.3)	129
Asian	3 (60.0)	2 (40.0)	5 (33.3)	10 (66.7)	20
Black	8 (25.8)	23 (74.2)	14 (36.8)	24 (63.2)	69
Other	53 (21.2)	197 (78.8)	174 (35.2)	321 (64.8)	745
Missing	0 (0.0)	5 (100.0)	1 (11.1)	8 (88.9)	14
IMD					
1	12 (18.8)	52 (81.3)	40 (32.3)	84 (67.7)	188
2	18 (31.6)	39 (68.4)	17 (25.4)	50 (74.6)	124
3	10 (24.4)	31 (75.6)	25 (32.9)	51 (67.1)	117
4	6 (15.8)	32 (84.2)	21 (33.3)	42 (66.7)	101
5	3 (13.0)	20 (87.0)	22 (41.5)	31 (58.5)	76
6	4 (23.5)	13 (76.5)	20 (43.5)	26 (56.5)	63
7	6 (20.7)	23 (79.3)	24 (43.6)	31 (56.4)	84
8	9 (32.1)	19 (67.9)	16 (30.8)	36 (69.2)	80
9	5 (20.8)	19 (79.2)	18 (39.1)	28 (60.9)	70
10	5 (18.5)	22 (81.5)	16 (34.0)	31 (66.0)	74
Month of sample collection					
October	1 (50.0)	1 (50.0)	0 (0.0)	9 (100.0)	11
November	0 (0.0)	1 (100.0)	9 (50.0)	9 (50.0)	19
December	2 (15.4)	11 (84.6)	22 (28.6)	55 (71.4)	90
January	9 (18.4)	40 (81.6)	45 (46.9)	51 (53.1)	145
February	18 (24.0)	57 (76.0)	50 (32.9)	102 (67.1)	227
March	32 (21.3)	118 (78.7)	56 (32.9)	114 (67.1)	320
April	10 (21.3)	37 (78.7)	21 (29.2)	51 (70.8)	119
May	6 (54.5)	5 (45.5)	16 (45.7)	19 (54.3)	46
Risk group					
Yes	34 (33.7)	67 (66.3)	156 (46.8)	177 (53.2)	434
No	21 (15.9)	111 (84.1)	46 (18.3)	205 (81.7)	383
Missing	23 (20.0)	92 (80.0)	17 (37.8)	28 (62.2)	160
Regions					
North	28 (19.6)	115 (80.4)	62 (36.9)	106 (63.1)	311
South	19 (21.6)	69 (78.4)	58 (36.7)	100 (63.3)	246
Midlands + East	25 (27.5)	66 (72.5)	76 (36.0)	135 (64.0)	302
London	6 (23.1)	20 (76.9)	23 (25.0)	69 (75.0)	118

The crude overall VE for all ages was 45.9% (95% CI 26.9–60.0) for all influenza types, which decreased to 33.4% (95% CI 2.3–54.6) after adjusting for age group, sex, IMD, ethnicity, region, month and risk group (Table 3).

Overall by route, the adjusted VE for all influenza types was 41.9% (95% CI 7.3–63.6) when administered intra-nasally (LAIV) and 28.8% (95% CI −31.1 to 61.3) when administered intra-muscularly (IIV) (Table 5).

**Table 5.** Number of hospitalised individuals positive (cases) and negative (controls) for influenza, by vaccination status and VE estimates by subtype and route in 2–16 year olds in 2015–2016, England

Influenza type	Cases (vac/unvac)	Controls (vac/unvac)	Crude VE (95% CI)	Adjusted VE^a^ (95% CI)
Any influenza				
Overall	78/270	219/410	45.9 (26.9–60.0)	33.4 (2.3–54.6)
Intra-nasal	46/270	127/410	45.0 (20.3–62.0)	41.9 (7.3–63.6)
Intra-muscular	27/270	78/410	47.4 (16.4–66.9)	28.8 (−31.1 to 61.3)
Influenza A				
Overall	48/148	219/410	39.3 (12.6–57.8)	31.3 (−9.9 to 57.1)
Intra-nasal	33/148	127/410	28.0 (−10.3 to 53.0)	27.9 (−22.6 to 57.6)
Intra-muscular	13/148	78/410	53.8 (14.5–75.1)	50.6 (−15.4 to 78.8)
Influenza B				
Overall	33/124	219/410	53.1 (28.2–69.3)	31.4 (−21.3 to 61.2)
Intra-nasal	14/124	127/410	63.6 (34.4–79.7)	61.0 (11.3–82.8)
Intra-muscular	16/124	78/410	32.2 (−20.4 to 61.8)	−13.8 (−160.0 to 50.2)
Influenza A(H1N1)pdm09				
Overall	34/120	219/410	47.0 (19.7–65.0)	40.3 (−2.9 to 65.4)
Intra-nasal	23/120	127/410	38.1 (−0.9 to 62.0)	42.4 (−7.8 to 69.2)
Intra-muscular	9/120	78/410	60.6 (19.1–80.8)	46.3 (−40.9 to 79.5)

a Adjusted VE by age group, sex, IMD, ethnicity, region, month and risk group.

By influenza sub-type, non-significant VE estimates were seen (Table 5). For influenza A(H1N1)pdm09 the overall estimate was 40.3% (95% CI −2.9 to 65.4), 42.4% (95% CI −7.8 to 69.2) for LAIV and 46.3% (95% CI −40.9 to 79.5) for IIV. For influenza B the overall estimate was 31.4% (95% CI −21.3 to 61.2), 61.0% (95% CI 11.3–82.8) for LAIV and −13.8% (−160.0 to 50.2) for IIV.

By the target age group, non-significant VE estimates were also seen. For the target age group for vaccination in the 2015–2016 season (2–6 year olds) the adjusted VE was 30.0% (95% CI −10.7 to 55.7) and for the non-target age group for vaccination (7–16 year olds) the adjusted VE was 45.6% (95% CI −17.6 to 74.8).

When comparing with no vaccination in both the current or previous influenza seasons, the adjusted VE for being vaccinated in both seasons was 50.8% (95% CI 18.2–84.1) (Table 6).

**Table 6.** Number of individuals positive (cases) and negative (controls) and VE estimates by prior vaccination history in 2–16 year olds in 2015–2016, England

Previous vaccination, any influenza type	Cases	Controls	Crude VE (95% CI)	Adjusted VE^a^ (95% CI)
Not vaccinated in either season	129	326	Baseline	Baseline
Vaccinated in 2014–2015, not 2015–2016	9	73	68.8 (35.9–84.9)	63.9 (18.2–84.1)
Vaccinated in 2015–2016, not 2014–2015	24	75	19.1 (−33.79 to 51.1)	2.6 (−73.3 to 45.2)
Vaccinated in both seasons	22	134	58.5 (31.9–74.7)	50.8 (18.2–84.1)
Missing	164	21		

a Adjusted VE by age group, sex, IMD, ethnicity, region, month and risk group.

## Discussion

The study assessed VE against hospitalisation during the 2015–2016 influenza season in England and found an overall significant VE of 33.4% against any influenza in children aged 2–16 years. The results indicate intranasal vaccine is likely to be effective. Risk factor was shown to be an important confounder in the analysis, which has often not been the case in studies looking at primary care end points [5, 6].

Overall VE was higher in children who received LAIV compared with IIV. By subtype, LAIV VE was slightly higher against B compared with influenza A(H1N1)pdm09, although these differences were not significant. The results in relation to prior vaccination are limited by small numbers however provide reassurance of the benefit of annual vaccination with evidence of significant protection if vaccinated in both seasons and possible cumulative effect.

Our findings of overall effectiveness against influenza-related hospitalisation in 2015–2016 in children are consistent with other published findings that influenza vaccination in 2015–2016 provided significant protection. In particular our study shows similar, although slightly lower (42% (95% CI 7.3–63.6) for all ages and 30.0% (95% CI −10.7 to 55.7) in the target age group for vaccination), estimates for LAIV to those found using the screening method against laboratory-confirmed influenza hospitalisation (54.5% (95% CI 31.5–68.4%)) [9] and in primary care (57.6% (95% CI 25.1–76.0%)) in England in 2015–2016 [5]. Internationally these results are also similar to those seen in Finland in primary care for 2 year olds in 2015–2016 [16]. They are however discordant with those seen in the United States where they found little evidence of effectiveness of LAIV in protecting children against laboratory-confirmed illness in primary care in 2015–2016 resulting in the removal of the recommendation to use LAIV [7, 8]. The reasons for these findings remain under investigation. Prior season vaccination has been hypothesised as a potential reason since the paediatric programme has been running for almost 10 years in the United States [17], however results from the UK [5] and Finland [16] as well as this study do not support this hypothesis.

The TNCC methodology has previously been used to assess the effectiveness of influenza vaccine in high-risk groups hospitalised in England against pandemic (H1N1) 2009 infection [18]. The test-negative design has a number of advantages; most notably is that both cases and controls should have a high degree of comparability, since they are recruited at the same time with the clinician not knowing the outcome of testing, thus reducing the risk of selection bias. Selection bias is also reduced by the fact that both cases and controls sought to care for similar sets of symptoms, reducing bias due to healthcare seeking behaviour which is in turn associated with vaccine uptake [19]. Despite this in studies using hospitalisation outcomes, the method may be subject to bias due to the fact that many underlying diseases increase the risk of hospitalisation for respiratory symptoms, but at the same time some of these diseases are indication for vaccination [20]. This is likely to explain the important confounding effect of risk-group when looking at severe end-points. The difference in the proportion of cases and controls that have a risk factor in this study is noteworthy. The limitations of this study include the fact that it had had limited power for the various stratifications. In addition, laboratory testing for influenza infection in the hospitalised age group studied tends to occur mainly among those presenting to secondary care. This will have a limited effect on the estimate of VE as cases and (test-negative) controls are likely to have similar severity of illness in order to be tested. Positivity rates between cases and controls differed significantly by month of sample collection despite group matching the controls to cases on week of sample. This is likely due to remaining differences from the return rates and missing data, as well as the fact the matching was carried out on the entire dataset prior to making any exclusions. As such age group and month were still included in the analysis.

This study provides evidence for the effectiveness of influenza vaccination in preventing hospitalisations due to influenza in children in 2015–2016 and continues to support the rollout of the LAIV childhood programme in England. The test-negative design is becoming increasingly popular for use in hospital-based studies adding to evidence of influenza VE in preventing severe influenza illness which is important to inform current vaccination strategies.
